# MAIT Cells at the Fetal-Maternal Interface During Pregnancy

**DOI:** 10.3389/fimmu.2020.01788

**Published:** 2020-08-19

**Authors:** Helen Kaipe, Johanna Raffetseder, Jan Ernerudh, Martin Solders, Eleonor Tiblad

**Affiliations:** ^1^Division of Biomolecular and Cellular Medicine, Department of Laboratory Medicine, Karolinska Institutet, Stockholm, Sweden; ^2^Department of Clinical Immunology and Transfusion Medicine, Karolinska University Hospital, Stockholm, Sweden; ^3^Department of Biomedical and Clinical Sciences, Linköping University, Linköping, Sweden; ^4^Department of Clinical Immunology and Transfusion Medicine, and Department of Biomedical and Clinical Sciences, Linköping University, Linköping, Sweden; ^5^Center for Fetal Medicine, Karolinska University Hospital, Stockholm, Sweden; ^6^Division of Clinical Epidemiology, Department of Medicine Solna, Karolinska Institutet, Stockholm, Sweden

**Keywords:** MAIT cells, placenta, pregnancy, decidua, intervillous blood

## Abstract

One of the main functions of the human placenta is to provide a barrier between the fetal and maternal blood circulations, where gas exchange and transfer of nutrients to the developing fetus take place. Despite being a barrier, there is a multitude of crosstalk between maternal immune cells and fetally derived semi-allogeneic trophoblast cells. Therefore, the maternal immune system has a difficult task to both tolerate the fetus but at the same time also defend the mother and the fetus from infections. Mucosal-associated invariant T (MAIT) cells are an increasingly recognized subset of T cells with anti-microbial functions that get activated in the context of non-polymorphic MR1 molecules, but also in response to inflammation. MAIT cells accumulate at term pregnancy in the maternal blood that flows into the intervillous space inside the placenta. Chemotactic factors produced by the placenta may be involved in recruiting and retaining particular immune cell subsets, including MAIT cells. In this Mini-Review, we describe what is known about MAIT cells during pregnancy and discuss the potential biological functions of MAIT cells at the fetal-maternal interface. Since MAIT cells have anti-microbial and tissue-repairing functions, but lack alloantigen reactivity, they could play an important role in protecting the fetus from bacterial infections and maintaining tissue homeostasis without risks of mediating harmful responses toward semi-allogenic fetal tissues.

## Introduction

During pregnancy, the maternal immune system is confronted with foreign antigens derived from the semi-allogenic fetus and placenta. A challenging task is therefore to display tolerance toward the HLA-disparate fetus and at the same time maintain anti-microbial responses. Feto-maternal tolerance is retained due to several mechanisms, including physical barriers, a diminished expression of polymorphic HLA molecules on fetal trophoblast cells, and production of immunosuppressive factors from fetally derived cells including trophoblasts, as well as maternally derived cells including both stromal cells and immune cells (1, 2). However, it is evident that the maternal immune system not only detects but also reacts toward fetal antigens. For instance, it has been shown that women during early pregnancy transiently increase T cell-mediated responses toward tumor-associated antigens that are highly expressed by fetal trophoblasts in the placenta, including HER2 and WT1 (3). Furthermore, fetal DNA and fetal immune cells are detected in the maternal circulation (4), and anti-HLA antibodies are often developed during pregnancy (5).

## Placental Structure and Fetal-Maternal Interface

**Figure 1:**
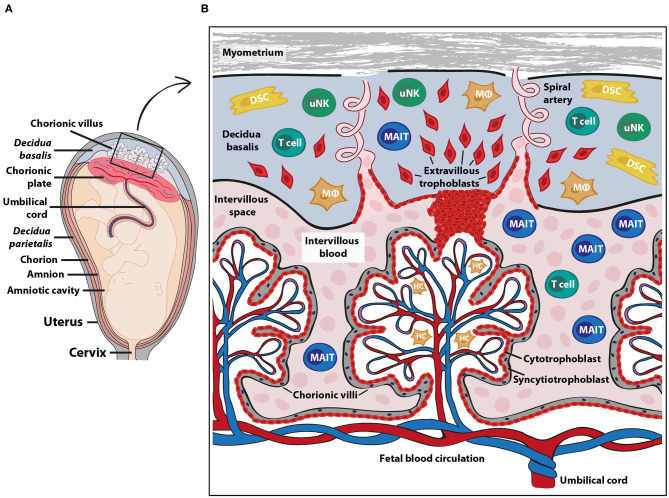
Fetal-maternal interface and immune cells at term pregnancy. The placenta serves to ensure exchange of nutrients and gases between the maternal and fetal blood circulation. The fetal part of the fetal-maternal interface consists of chorionic villi that extend from the chorionic plate **(A)** into the intervillous space and bathe in maternal intervillous blood **(B)**. On the maternal side, the decidua parietalis and decidua basalis are in direct contact with fetal membranes (amniochorion) and the invading fetal extravillous trophoblasts, respectively **(A)**. The intervillous blood enters the intervillous space through spiral arteries **(B)** and leaves this compartment through uterine veins (not shown). Maternal immune cells in the intervillous blood are in direct contact with the fetal syncytiotrophoblast and decidual immune cells can interact with extravillous trophoblasts **(B)**. DSC, decidual stromal cells; HC, Hofbauer cells (fetal macrophages); MAIT, mucosal associated invariant T cells; MΦ, macrophages; uNK, uterine natural killer cells. The pink cells in the intervillous space depict erythrocytes.

A main function of the placenta is to provide the developing fetus with nutrients and gas exchange through an intricate placental blood circulation system. The maternal placental circulation is gradually established during the first trimester (6), and from the second trimester until birth, maternal arterial blood delivers oxygen, IgG antibodies and nutrients over a thin membrane of fetally derived cells to the fetal blood circulation via the umbilical cord (7) (Figure 1A). Maternal immune cells are in close contact with semi-allogeneic fetal trophoblast cells in two anatomically different parts of the placenta; in the decidua and in the intervillous space (Figure 1B). The decidua is a specialized tissue emanating from the uterine endometrium, which functions to prepare for and accommodate pregnancy. The decidua is invaded by both maternal immune cells and fetal extravillous trophoblasts during early pregnancy. The extravillous trophoblasts play an important role in the remodeling of the spiral arteries (8), thereby securing the maternal blood flow into the intervillous space from the second trimester, where nutrients and gas exchange to the fetus takes place over the syncytiotrophoblast layer of the chorionic villi (Figure 1B).

## Maternal Immune Cells at the Fetal-Maternal Interface

The composition of maternal immune cells in the decidua has been characterized both in early and term pregnancy, showing that first trimester decidua is dominated by CD56^bright^ NK cells with few T cells, whereas the proportion of T cells increases substantially at term (9). Macrophages in the decidua maintain their proportion during pregnancy and display immune regulatory actions (10). Maternal decidual stromal cells are tissue resident cells that can suppress immune activation (11, 12). It has been suggested, based on murine studies, that these cells prevent activated maternal T cells from entering the decidua in early pregnancy by silencing of the T cell-attracting chemokines CXCL9 and CXCL10 (13). In humans, the mechanisms for the relative low proportion of T cells in the first trimester decidua are not known. Regulatory T cells are enriched in the decidua (14), and both γδ T cells and CD8^+^ T cells make up larger portions relative to CD4^+^ T cells compared with blood (2). γδ T cells have been suggested to have a protective role during early pregnancy by producing IL-10 and promote trophoblast survival and invasion (15). Invariant NKT cells are also enriched in the decidua relative to peripheral blood (16). Activation of NKT cells with the CD1d agonist αGalCer promotes pregnancy loss in murine models (17). Single cell analysis of the early fetal-maternal interface in the decidua has further identified predicted regulatory interactions between maternal immune cells and fetal trophoblasts that prevent harmful immune reactions (18). For instance, extravillous trophoblasts highly express the genes encoding PD-L1 and CD155, which could inhibit cytotoxic responses by T cells and NK cells via PD-1 and TIGIT ligation, respectively.

In contrast to the decidua, very little is known about the composition and function of maternal immune cells in the intervillous space, in which fetal villous tissue bathes in maternal blood (Figure 1B). The general notion has been that the blood volume in the intervillous space is replaced 2–3 times every minute to provide gas exchange (7), suggesting that the intervillous blood cell composition reflects that of peripheral blood. However, others (19, 20) and our own recent studies (21–23) show that NK cells and certain T cell and B cell subsets are enriched in the intervillous blood, indicating that particular cell types are sequestered in the intervillous space, which is discussed in more detail below. Similar to the spleen and liver, in which a proportion of the circulating blood is shunted into the low-pressure pools in the sinusoids, it is likely that maternal blood constituents entering the intervillous space are retained inside the placenta. Mucosal-associated invariant T (MAIT) cells are one type of immune cell subset that is relatively enriched in intervillous compared to peripheral blood at term pregnancy (21, 22) and MAIT cells are also present in decidual tissues (24).

## Mait Cells At the Fetal-Maternal Interface and in Uterine Endometrium

In contrast to conventional T cells, which need to get their peptide antigen presented on highly polymorphic MHC molecules, MAIT cells are restricted to the monomorphic MHC-like receptor 1 (MR1) molecule (25). MAIT cells express the semi-invariant T cell receptor alpha chain Vα7.2 (TRAV1-TRAJ33) (26), and respond to vitamin B2 metabolites in an MR1-dependent manner (27). These non-peptide ligands are produced by microbes with a functional riboflavin biosynthesis pathway (27–31), including many commensal and pathogenic bacterial and fungal species. Inflammatory cytokines, such as IL-12 and IL-18, can also partially activate MAIT cells without the need for TCR-ligation (32), which broadens their capacity to also be involved in anti-viral and inflammatory responses (33). The majority of MAIT cells are CD8^+^, but a subset of MAIT cells lacks the expression of both CD4 and CD8 (double-negative, DN), and a minor fraction expresses CD4 (34–36). MAIT cells display a memory phenotype and they respond quickly by producing cytotoxic molecules and inflammatory cytokines upon activation. Moreover, there is emerging evidence suggesting that MAIT cells also express tissue repair signatures upon TCR-ligation (37–40). Thus, MAIT cells are anti-microbial and tissue-repairing T cells that lack the capacity to respond to allogeneic HLA molecules, which could be ideal traits of effector cells at the fetal-maternal interface. On the other hand, since MAIT cells respond quickly and are capable of inducing prominent inflammation, they must also be kept under strict control.

In healthy term pregnancies, pregnant women had lower proportions of MAIT cells in the circulation compared to non-pregnant women, suggesting that MAIT cells home to the placenta (22). Indeed, the proportion of MAIT cells among both CD3^+^ T cells and total CD45^+^ cells is approximately 2-fold higher in placental intervillous blood compared to peripheral blood in healthy term pregnancies (21). Ravi et al. showed that proportions of peripheral MAIT cells were unaltered during the course of pregnancy (41), and since MAIT cell proportions were not investigated before pregnancy it can be speculated that MAIT cells localize to the placenta or other tissues already during early gestation. Although leukocyte counts increase during pregnancy, the lymphocyte concentration shows a slight decrease from early to late pregnancy, which can be attributed to the hemodilution that takes place because of the physiological increase in plasma volume during pregnancy (42). However, since the proportion of T cells in blood does not change during pregnancy (43) and since most studies report MAIT cell frequencies as proportion of CD3^+^ T-cells, early and late pregnancy can safely be compared.

Intervillous MAIT cells exhibit a stronger IFN-γ and granzyme B expression compared to paired peripheral MAIT cells in response to riboflavin-producing *Escherichia coli* (21). However, in a resting state, intervillous and peripheral MAIT cells express similar levels of the activation markers HLA-DR and CD69, while intervillous MAIT cells express lower levels of CD25 and PD-1 (21, 22). Intervillous MAIT cells consist of a higher proportion of DN MAIT cells compared to peripheral MAIT cells. This is in accordance with the reported decrease in peripheral DN MAIT cells from the first to the third trimester (41), which could potentially reflect their localization to the intervillous space of the placenta. Together, these data suggest that maternal MAIT cells in the intervillous space of the placenta at term pregnancy display an increased inflammatory response to riboflavin-producing bacteria and that there are phenotypic differences between peripheral and intervillous MAIT cells. It remains to be determined if the elevated inflammatory response of intervillous MAIT cells is due to intrinsic properties or whether extrinsic effects, such as antigen presentation or soluble factors, are involved in potentiating the response compared to peripheral MAIT cells.

MAIT cells are present also in the endometrium and cervix of the genital tract of non-pregnant women, but the endometrium contains lower frequencies of MAIT cells out of CD3^+^ T cells compared to peripheral blood (44). After fertilization, the endometrium undergoes decidualization to form the decidua. The part of the decidua underlying the placental disc, which is perfused by spiral arteries to provide the intervillous space with maternal blood, is termed decidua basalis (Figures 1A,B). The decidua parietalis refers to the decidual layer that is attached to the fetal membrane, consisting of the fused chorion and amnion which create the amniotic sac. For early pregnancy, it is known that MAIT cells are present in the decidua (18), but there is no information on their relative abundance, phenotype or location. In contrast to the non-pregnant endometrium, which contains fewer MAIT cells compared to peripheral blood, the proportion of MAIT cells in term pregnancy decidua parietalis is similar to peripheral MAIT cells, and MAIT cells are even more abundant in the decidua basalis compared to the decidua parietalis (24). This may suggest that MAIT cells to some degree home to the decidual mucosa at term pregnancy.

Decidual MAIT cells at term express high levels of CD69, consistent with a tissue-residency phenotype (21, 24). MAIT cells in decidua parietalis express higher levels of PD-1, CD38 and CD25 compared to MAIT cell in decidua basalis, indicating a more activated phenotype (24). While endometrial MAIT cells are biased toward IL-17 and IL-22 expression, with less production of IFN-γ and granzyme B (44), decidual MAIT cells produce higher levels of granzyme B and similar levels of IFN-γ in response to *E. coli* as compared to peripheral MAIT cells (21). It is not yet known if decidual MAIT cells also have a propensity to produce IL-17 and IL-22. Mucosal production of IL-17 and IL-22 is important for anti-bacterial and anti-fungal responses and mucosal barrier function, respectively (45, 46), suggesting that it would be an advantage also for decidual MAIT cells to possess this function. It remains to be determined how decidual MAIT cells in early and late pregnancy are polarized in terms of cytokine production, but in contrast to genital tract MAIT cells it appears that IFN-γ and cytotoxic molecule secretion from term decidual MAIT cells are comparable to that of peripheral MAIT cells, indicating that pregnancy may affect the functional responses of uterine MAIT cells.

## Chemokine-Induced Attraction Of Mait Cells to The Placenta

Intervillous MAIT cells do not express the proliferation marker Ki67, suggesting that they are in a non-cycling state (21). It can therefore be speculated that the increased proportion of MAIT cells in the intervillous space is due to recruitment and retention by chemotactic factors. The fetal placenta and its trophoblasts produce a wide array of chemokines (22, 47), and maternal platelets in the intervillous space may also contribute to local chemokine release (48). Interestingly, the chemokine pattern in intervillous plasma is clearly different compared to paired peripheral plasma, with higher levels of several chemokines, including macrophage migration inhibiting factor (MIF), CCL2, CCL25, CXCL9, and CXCL10 (22). Other chemokines are instead lower in intervillous compared to peripheral plasma, including CCL21 and CCL27 (22). MAIT cell proportions in intervillous blood and in decidua are positively associated to levels of MIF and CCL25 in intervillous plasma. Migration assays have shown that conditioned medium from term fetal placental tissues attracts effector memory T cells in general and MAIT cells in particular, and that MIF is one of the factors involved in attracting MAIT cells (22). MIF is a chemokine-like cytokine that binds to CXCR4 and CXCR2 (49). MAIT cells express high levels of CXCR4 but low levels of CXCR2 (22, 34), suggesting that CXCR4 is an important receptor for MIF-mediated homing of MAIT cells. CD8^+^ and DN MAIT express similar proportions of CXCR4 (50), and both subsets migrated to the same extent toward placental conditioned medium (22). However, CD8^+^ MAIT cells have been described to express higher levels of CCR6 compared to DN MAIT cells (51).

In contrast to MAIT cells, proportions of conventional CD8^+^ effector memory T cells, which are also enriched in intervillous blood of term placentas, showed no correlation to MIF levels but to the CXCR3-ligands CXCL9, CXCL10, and CXCL11 in intervillous plasma (22). Moreover, the levels of the CCR6-ligand CCL20 is correlated to proportions of mature naïve B cells in intervillous blood (23). This suggests that different kinds of chemokines are involved in attracting and retaining distinctive immune cell subsets to the placenta, but it is likely a combination of different chemokines that shapes the composition of immune cell subsets in the intervillous space. It should also be noted that other chemokines could play a more prominent role in attracting MAIT cells to other types of tissues. For instance, MAIT cells have been suggested to home to ascites in liver cirrhosis patients by CXCR3-CXCL10 ligation (52) and to the liver by CXCR6 and CCR6 and their ligands CXCL16 and CCL20, respectively (53).

Interestingly, both syncytiotrophoblasts and extravillous trophoblasts highly express the chemokine decoy receptor D6/ACKR2, which can decrease chemokine availability to control leukocyte migration (54). D6 internalizes and degrades inflammatory CC chemokines which are ligands to the classical chemokine receptors CCR1-CCR5 (55). It can be speculated that this atypical chemokine receptor with CC chemokine scavenging function can play a role in regulating the number and position of maternal immune cells at the fetal-maternal interface. It remains to be determined if D6 is involved in shaping the immune cell composition with increased accumulation of MAIT cells, effector memory T cells and mature naïve B cells in the intervillous space.

## Antigen-Presenting Molecules on Cells in the Placenta

The syncytiotrophoblasts, which line the fetal villi and are in immediate contact with maternal blood in the intervillous space (Figure 1B), appear to lack expression of the MR1 molecule both at early second trimester and at term (21). This is in line with the absence of HLA molecule expression on syncytiotrophoblasts from the second trimester (56). Thus, this lack of antigen presenting molecules could prevent any MR1- or HLA-mediated cytotoxicity toward the fetal syncytiotrophoblasts by maternal MAIT cells and T cells, respectively. It is not yet known if extravillous trophoblasts express MR1, but they do express HLA-C and the non-classical oligomorphic HLA-E, HLA-G and HLA-F at varying intensities during gestation (56). Extravillous trophoblasts also express CD1d in early pregnancy, suggesting that NKT cells may interact with fetal cells in the decidua (16). Fetal macrophages (also called Hofbauer cells) in the fetal villi (Figure 1B) express MR1 both at second trimester and at term, indicating that they can function as antigen-presenting cells to MAIT cells if the barrier of the fetal syncytium is broken (21). CD8^+^ maternal T cells can be detected inside the villi in villitis of unknown origin, a non-infectious condition that is associated with fetal growth restriction (57). Whether maternal MAIT cells are present in the villi during this condition is not yet known. MR1^+^ cells are also detected in term decidua and some of these cells are macrophages, as assessed by CD68 expression (21), indicating that decidual macrophages have the potential to present antigens to MAIT cells.

## Can Mait Cells Encounter Their Antigens in the Placenta?

It has long been thought that the placenta is devoid of microbes in healthy pregnancies. This perception was challenged by studies suggesting that the placenta has its own microbiome (58, 59), but emerging evidence indicates that the detection of the placental microbiome may have been caused by contamination during the analysis process (60–62). However, De Goffau et al. observed that approximately 5% of placentas contained *Streptococcus agalactiae*, which was concluded to not be due to contamination (63). *S. agalactiae* is associated with commensal carriage, but is also a neonatal pathogen since it can cause neonatal sepsis (64). *S. agalactiae* strains possess riboflavin operons (65), suggesting that they have the potential to produce MR1-ligands and act as MAIT cell targets. Seferovic et al. used 16S *in situ* hybridization to visualize bacteria in healthy placental tissues and found that microbes were present at low abundance and preferably were localized to the villous parenchyma and syncytiotrophoblast layers. Thus, the current literature suggests that bacterial cells occasionally are present in the healthy placenta. Intervillous and decidual MAIT cells could play a role in preventing bacteria from crossing the fetal-maternal barrier.

## Tissue-Repairing Capacity of Mait Cells

Apart from mediating pro-inflammatory responses upon infection, MAIT cells have recently been described to express a functional gene signature of tissue repair (37–40) and to have tissue protective capacities in murine models of inflammation (66, 67). The tissue repair function of MAIT cells is dependent on TCR-triggered activation, indicating that activation of this pathway is dependent on the presence of riboflavin-producing bacteria. The activating bacterial MR1-ligand 5-OP-RU can cross epithelial barriers (68), and it could potentially be present in organs devoid of infection, including the placenta. It is possible that intervillous MAIT cells are involved in maintaining barrier integrity to protect the fetal villi from barrier disruption and other placental lesions. Discontinuities in the syncytiotrophoblast layer with fibrin deposits are common in term villi (69). Fetal macrophages have been described to aggregate around injured regions of villous tissue in *ex vivo* models (70) and could potentially interact with maternal intervillous MAIT cells to assist tissue repair. Since MAIT cells express several genes encoding proteins involved in tissue repair and fibrin formation, including thrombospondin-1, furin and thrombin receptors (37, 38, 40), it is possible that they play a role in repairing the syncytiotrophoblast layer. It can also be speculated that MAIT cells could be involved in accelerating wound repair when the placenta is detached from the uterine wall and the spiral arteries are disrupted at birth. However, further studies are needed to increase our knowledge in the intriguing area of MAIT cells and tissue repair.

## Mait Cells in Pregnancy Complications

An insufficient invasion of extravillous trophoblasts leads to a poor development of spiral arteries and, hence, to an impaired maternal blood circulation in the intervillous space. This is one of the causal factors of preeclampsia. Preeclampsia affects 3–5% of pregnant women and is a leading cause of maternal and perinatal morbidity and mortality worldwide (71). Immunological factors are likely involved in the pathogenesis of preeclampsia, including an imbalance in CD4^+^ T cell subsets with increased proportions of inflammatory Th17 cells and less regulatory T cells (72) and elevated systemic inflammation (73), but the mechanisms remain to be defined. It was recently shown that the proportion of peripheral MAIT cells was lower in mothers with early-onset preeclampsia compared to healthy pregnancies (74). No investigation of placental MAIT cells was performed, and a low MAIT proportion in peripheral blood could account for homing to tissues as discussed above. Peripheral MAIT cells from preeclampsia patients also displayed a lower expression of PD-1, but higher expression of CD69 and perforin, compared to healthy pregnancies. Whether MAIT cells play an active role in preeclampsia remains to be determined.

Spontaneous preterm birth (PTB), i.e., birth before gestational week 37, is one of the leading causes of childhood morbidity and mortality. Similar to preeclampsia, there is an association with immunological factors also in PTB (75). For instance, maternal T cell infiltration is observed in chronic chorioamnionitis, the most common placental lesion leading to late spontaneous PTB, and increased influx of cytotoxic effector memory cells has been associated with preterm labor and birth (76). Although the overall frequencies of peripheral MAIT cells were unaltered during the course of healthy pregnancy, in HIV-infected pregnant women, and in women with subsequent PTB, MAIT cells subsets were altered with a higher proportion of CD8^+^ MAIT cells in first trimester in women with PTB compared to term birth (41). HIV-infection, which entails a higher risk of PTB, was associated with a higher proportion of CD8^+^ MAIT cells compared to HIV-negative women. In healthy pregnancies the proportion of CD8^+^ MAIT cells increased during the course of pregnancy. Functional differences in MAIT cell subsets have been described (51, 77), and CD8^+^ MAIT cells express more IFN-γ, granzyme B and perforin compared to DN MAIT cells (77) and CD4^+^ MAIT cells (51). It is possible that an imbalance in the different MAIT cell subsets during early pregnancy could be involved in immunological aberrations associated with PTB, but the putative importance of MAIT cells and different subsets of MAIT cells in PTB and other pregnancy complications still needs to be defined.

## Concluding Remarks

Several questions remain regarding the function of MAIT cells during pregnancy and the data available so far derive solely from observational studies on human pregnancies. It is not known if MAIT cells are enriched in the placenta throughout pregnancy or if they are retained in the intervillous space only at term. Selective enrichment of MAIT cells in the intervillous space but not in adjacent decidual tissue signify separate immunological entities which deserve more attention in future research. The enhanced functional response of term placental MAIT cells could indicate a putative role in placental inflammation and dysregulated MAIT cell responses could be involved in pregnancy complications. However, since MAIT cells have anti-microbial and tissue-repairing functions, but lack alloantigen reactivity, they could play an important role in protecting the fetus from bacterial infections and maintaining homeostasis at the fetal-maternal interface.

## Author Contributions

JR prepared the figure. All listed authors made a substantial intellectual contribution and approved the manuscript for publication.

## Conflict of Interest

The authors declare that the research was conducted in the absence of any commercial or financial relationships that could be construed as a potential conflict of interest. The handling editor declared a past co-authorship with one of the authors HK.
